# Observations of the Fine Structural Changes Associated with Merogony and Gametogony in *Eimeria necatrix* and Localization of Two Gametocyte Proteins

**DOI:** 10.3390/microorganisms13051135

**Published:** 2025-05-15

**Authors:** Yu Zhu, Dandan Liu, Lele Wang, Qianqian Feng, Feiyan Wang, Nianyu Xue, Zhaofeng Hou, Jinjun Xu, Junjie Hu, Jianping Tao

**Affiliations:** 1 College of Veterinary Medicine, Yangzhou University, Yangzhou 225009, China; zy18362822874@163.com (Y.Z.); ddliu@yzu.edu.cn (D.L.); 18252736323@163.com (L.W.); qianqianfeng98@163.com (Q.F.); dx120210181@stu.yzu.edu.cn (F.W.); yznianyuxue@126.com (N.X.); zfhou@yzu.edu.cn (Z.H.); jjxu@yzu.edu.cn (J.X.); 2 Jiangsu Co-Innovation Center for Prevention and Control of Important Animal Infectious Diseases and Zoonoses, Yangzhou University, Yangzhou 225009, China; 3 School of Ecology and Environmental Sciences, Yunnan University, Kunming 650091, China; jjhu@ynu.edu.cn

**Keywords:** *Eimeria necatrix*, coccidia, merogony, gametogony, electron microscopy, gametocyte protein, immunolocalization

## Abstract

Coccidian parasites possess complex life cycles involving asexual proliferation followed by sexual development, producing oocysts that are transmitted from host to host through feces, guaranteeing disease transmission. *Eimeria necatrix* is a highly pathogenic coccidian causing high mortality in birds. This study examined ultrastructural changes occurring during the third merogony, microgametogenesis, and macrogametogenesis of *E. necatrix*. The third-generation meront contained eight merozoites, each with coccidian-specific features like conoid, rhoptries, micronemes, and dense granules. Microgametes had a nucleus, mitochondrion, two flagella, and a basal apparatus. Macrogametes surrounded by two membranes (M1 and M2), contained organelles like WFB1, WFB2, endoplasmic reticulum, mitochondria, and tubular structures. Oocyst wall formation began with M2 separating from M1 and forming a loose veil around the organism. The WFB1 fused together to form the outer layer of the oocyst wall between M1 and M2, while M4 formed beneath M1. The WFB2 fused with the M4 to discharge its contents external to M4, which fused together to form the inner layer of the oocyst wall. Immunogold electron microscopy co-localization result showed that EnGAM22 localized to WFB1 and the outer wall, while EnGAM59 localized to WFB2 and the inner wall, suggesting they are key structural components of the oocyst wall.

## 1. Introduction

Chicken coccidiosis is a parasitic disease caused by the genus *Eimeria*, bringing huge economic losses to the poultry industry worldwide [1]. *Eimeria* spp. possess a complex life cycle involving merogony (or schizogony) and gametogony within the host and sporogony in vitro [2,3]. During gametogony, macrogamete and microgamete is fertilized to form zygotes; subsequently, an oocyst wall is formed surrounding the zygotes and then developed into oocysts. After being excreted with feces, unsporulated oocysts undergo sporogony in an extrahost environment, forming infectious sporulated oocysts that enable the disease to spread among hosts [2,3,4]. Therefore, understanding the molecular foundation and mechanisms of coccidian oocyst wall formation will help in identifying novel targets for the prevention and treatment of chicken coccidiosis.

The coccidian oocyst wall is a bilayered structure formed from the contents of two specific organelles, types 1 and 2 wall-forming bodies (WFB1 and WFB2, respectively), found exclusively in the macrogametocyte stage of coccidian parasites [5,6]. The oocyst wall consists mainly of proteins and lipids [7]. Gametocyte proteins are precursors of the oocyst wall proteins [8]. To date, three groups of gametocyte proteins have been confirmed to participate in *Eimeria* oocyst wall formation, including tyrosine-rich glycoproteins that localize to the WFB2 of macrogametocytes and the inner wall of *Eimeria* oocysts [6,9], the histidine–proline-rich protein that localizes to the WFB1 of macrogametocytes and the outer wall of the *Eimeria* oocyst [9], and cysteine-rich proteins (namely oocyst wall protein, OWP) that localize to WFB1 and the outer wall of the *Eimeria nieschulzi* oocyst [10]. Immunization with the purified WFB antigens could result in the production of parasite-specific IgG antibodies, which block parasite development and oocyst shedding, thus preventing the transmission of the disease [11,12].

*Eimeria necatrix* is a highly pathogenic coccidium and can cause high mortality in susceptible birds, particularly in chickens over 8 weeks of age when raised on a litter floor [3]. Unlike other species of avian coccidium, whose merogony and gametogony occur in the same segment of the intestine, first- and second-generation meronts of *E. necatrix* are primarily located within the mid-intestinal area of host chickens, and third-generation meronts (Mer-3) and later oocyst development occur only in the caecum [3]. The ultrastructure of *E. necatrix* is similar to other *Eimeria* parasites [13,14]. In a previous study using an indirect immunofluorescence assay and laser confocal microscopy, we found that *Eimeria necatrix* gametocyte proteins 22 and 59 (EnGAM22 and EnGAM59) localized to WFB1 and WFB2 and were involved in the formation of the outer and inner layers of the oocyst wall of *E. necatrix*, respectively [9]. In the present study, Mer-3, third-generation merozoites (MZ-3), and the gametogony of *E. necatrix* were observed by transmission electron microscopy (TEM), and the ultrastructural localizations of EnGAM22 and EnGAM59 proteins in the gametocytes and unsporulated oocysts of *E. necatrix* were confirmed using the immunogold electron microscopic co-localization technique to clearly define the function of EnGAM22 and EnGAM59 proteins. This basic knowledge about the fine structure and development of *E. necatrix* may represent targets for avian coccidiosis control.

## 2. Materials and Methods

### 2.1. Parasites and Animals

The *Eimeria necatrix* (Yangzhou strain) used in this study was originally isolated from chickens that died from *E. necatrix* infection in 2009, obtained from the Key Laboratory for Avian Preventive Medicine at Yangzhou University (48 East Wenhui Road, Yangzhou, China), as confirmed by microscopic examination and sequence analysis of the internal transcribed spacer region of genomic DNA [15]. This strain has been maintained in our laboratory. The oocysts were periodically propagated in 3–4-week-old chickens. Oocysts were isolated and harvested from feces by salt flotation and centrifugation, sporulated in vitro at 28 °C, and stored in 2.5% potassium dichromate solution at 4 °C [15].

One-day-old chickens, purchased on the day of hatching from the Poultry Institute of Chinese Academy of Agricultural Sciences (Yangzhou, China), were housed in *Eimeria*-free isolation cages and were provided with complete feed and clean water without anticoccidial drugs or antibiotics. Prior to inoculation, chicken feces were examined using salt flotation and light microscopy to confirm the absence of *Eimeria* infection.

All animal care and procedures were conducted according to the guidelines for animal use in toxicology. All study protocols were approved by the Animal Care and Use Committee of the College of Veterinary Medicine, Yangzhou University (Approval ID: SYXK [Su] 2021-0027, Date: 26 March 2021).

### 2.2. Polyclonal Antibodies

Rabbit anti-rEnGAM22 and mouse anti-rEnGAM59 polyclonal antibodies (pAbs) were obtained using a previously published method [9]. Antibody levels were determined using an enzyme-linked immunosorbent assay (ELISA) method as described in a previous study [15].

### 2.3. Tissue Processing

Five-week-old chickens were orally infected with approximately 3.8 × 10^4^ sporulated oocysts of *E. necatrix*. At 148 h post infection (hpi), the chickens were euthanized, and then ceca were removed and washed with cold PBS (137 mM NaCl, 2.7 mM KCl, 10 mM Na_2_HPO_4_·12H_2_O, 2 mM KH_2_PO_4_, pH 7.4). The cecum tissues were immediately fixed using the specialized fixation solution and were then cut into about 1 mm^3^ tissue blocks using a surgical blade. Subsequently, these tissue blocks were transferred to an Eppendorf tube containing a fresh fixation solution for fixation at 4 °C. After fixation, the tissue blocks were dehydrated through a graded ethanol series (30, 50, 70, 80, 85, 90, 95, and 100%) and were then embedded in resin. After polymerizing in a low-temperature UV polymerizer (Electron Microscopy, Beijing, China) for about 48 h at −20 °C, the resin blocks were taken out for future use.

### 2.4. Transmission Electron Microscopy Observation

The resin blocks were cut to 70–80 nm thickness using an ultra-microtome (Leica UC7, Wetzlar, Germany), and ultrathin sections were fished out onto 150-mesh nickel grids with formvar film. The nickel grids with the sections were suspended on ultrapure water for 5 min and were then rinsed three times for 5 min each time with Tris-buffered Saline (TBS; 10 mM Tris, 150 mM NaCl, 20 mM NaN_3_; pH 8.0). Then, the sections were stained with 2% uranium acetate-saturated alcohol solution for 8 min in the dark and then rinsed three times for 15 min each time with 70% ethanol. After rinsing three times for 15 min each time with ultrapure water, the nickel grids with the sections were dried on filter paper, and then dried in grids board at 37 °C for 10 min. The samples were observed using TEM (Hitachi HT7800, Tokyo, Japan).

### 2.5. Immunoelectron Microscopy Observation

After rinsing with TBS, the sections were blocked with 1% bovine serum albumin (BSA) in TBS for 30 min at room temperature and were then incubated with rabbit anti-rEnGAM22 pAb (1:200 dilution) and mouse anti-rEnGAM59 pAb (1:200 dilution) overnight at 4 °C. After washing with TBS three times for 15 min each time, the sections were incubated with 4 nm golden particles labeled goat anti-rabbit secondary antibody (Sigma-Aldrich, St. Louis, MO, USA) and 12 nm golden particles labeled goat anti-mouse secondary antibody (Sigma-Aldrich) for 20 min at room temperature, then for 1 h at 37 °C, and finally for 0.5 h at room temperature. The sections were observed using TEM (Hitachi HT7800, Tokyo, Japan).

## 3. Results

### 3.1. Development and Ultrastructure of Third-Generation Meronts and Merozoites

**Figure 1:**
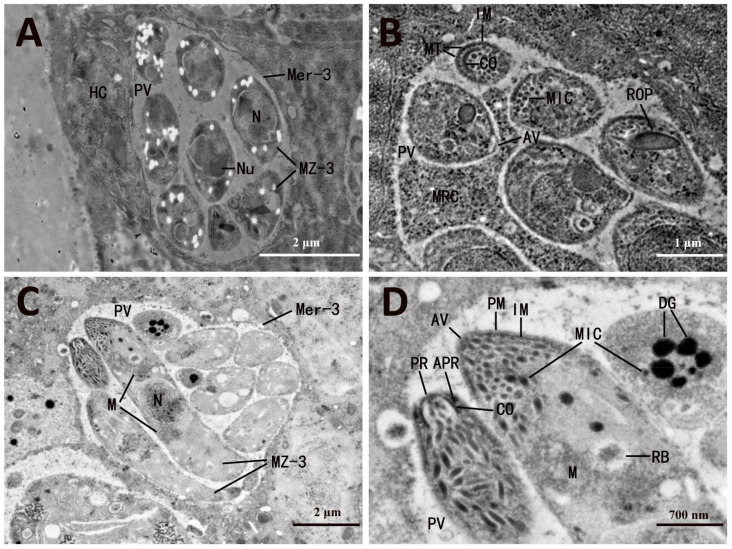
Electron micrographs of third-generation merogony and merozoites of *Eimeria necatrix*. (**A**) Later-stage Mer-3 with several young MZ-3 located in PV. (**B**) Slightly later-stage Mer-3 with MRC showing AV, IM, MT, CO, MIC, and ROP. (**C**) Matured Mer-3 with mature MZ-3, showing N, M, and PV. (**D**) Enlargement of the section displayed in (**C**) showing PM, IM, PR, APR, CO, AV, MIC, M, DG, and RB. Mer-3, third-generation meront; MZ-3, third-generation merozoites; MRC, meront residual cytoplasm; PV, parasitophorous vacuole; AV, anterior vesicle; IM, inner membrane; MT, subpellicular microtubule; CO, conoid; MIC, microneme; ROP, rhoptry; N, nucleus; Nu, nucleolus; M, mitochondria; PM, plasma membrane; PR, pre-conoidal ring; APR, anterior polar ring; DG, dense granule; RB, refractile body.

Second-generation merozoites of *E. necatrix* invade the cecal epithelial cells of chickens and develop into third-generation meronts (Mer-3) and merozoites (MZ-3). Mer-3 was parasitized in the parasitophorous vacuole (PV) (Figure 1). The slightly later-stage Mer-3 of *E. necatrix* contained eight MZ-3 (Figure 1A) and one meront residual cytoplasm (Figure 1B), and MZ-3 was released from the meront residual cytoplasm (Figure 1B). As the meronts matured, the meront residual cytoplasm disappeared (Figure 1C). The MZ-3 was bounded by a pellicle consisting of a plasma membrane and an inner membrane, and the subpellicular microtubules were uniformly arranged at equal intervals underneath the inner membrane (Figure 1B). An anterior vesicle was dome shaped and located at the apex of MZ-3 (Figure 1B,D). A pre-conoidal ring localized at the front end of MZ-3, and an anterior polar ring was located on the outer side of conoid (Figure 1D). MZ-3 contained a nucleus with a nucleolus and peripheral chromatin and an apical complex consisting of the conoid, rhoptries (ROP), and micronemes (MIC) (Figure 1C,D). The front end of ROP was filiform, whereas its posterior region was wider and club shaped (Figure 1B). MICs were rod-shaped granules localized at the apical end of MZ-3 (Figure 1C,D). In addition, MZ-3 had several mitochondria around the nucleus and a refractile body and several dense granules in the cytoplasm (Figure 1C,D).

### 3.2. Microgametocyte Ultrastructure and Microgamete Formation

**Figure 2:**
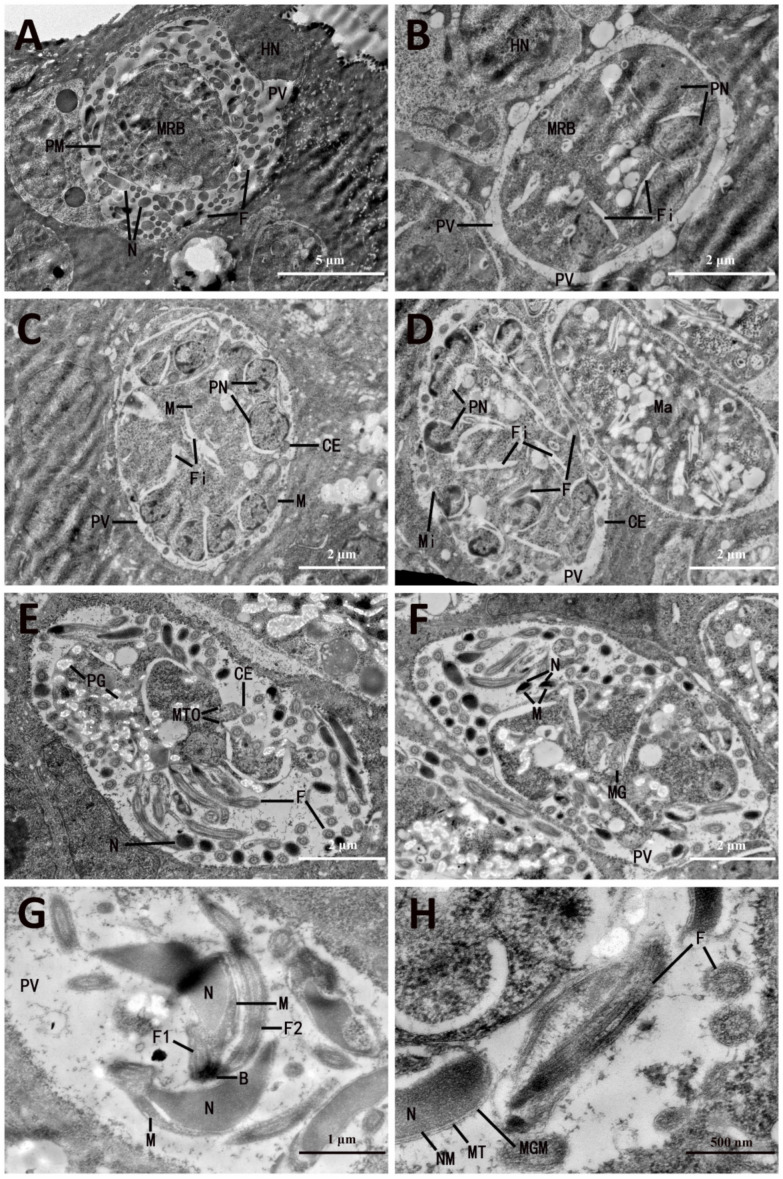
Electron micrographs of microgametocytes and microgametes of *Eimeria necatrix*. (**A**) A middle stage of Mi located under HN, with several young MG scattered in PV. (**B**) An early stage of Mi with several Fi that appeared in cytoplasm. (**C**) An early stage of Mi located in PV, with several mitotic PN and CE distributed in the periphery of the cytoplasm. (**D**) An early stage of Mi with several F and more Fi appearing in cytoplasm. (**E**) An old stage of Mi with numerous PG inside surrounded by many mature MG and F. (**F**) An old stage of Mi showing N and M in MG. (**G**) The portrait section of the head of MG showing N, M, and two F. (**H**) The N of mature MG are surrounded by NM, MT, and MGM, and the F contains an MT-typical 9 + 2 structure of Fi. PV, parasitophorous vacuole; PN, plasma nucleus; CE, centriole; M, mitochondria; Ma, macrogametocyte; Mi, microgametocyte; MG, microgamete; N, nucleus; HN, host nucleus; MRB, microgametocyte residual body; PM, plasma membrane; F, flagella; MT, microtubules; PG, polysaccharide granules; MTO, underlying cylinder-like osmiophilic layer at places where the microgametes are pinched off; B, basal apparatus; NM, nucleus membrane; MGM, microgamete membrane; Fi, fissure.

At 148 hpi, microgametocytes were seen at various stages of development. The microgametocytes inside PV developed below the nucleus of epithelial cells of the cecum (Figure 2A), and the microgametocyte itself was surrounded by a unit membrane (Figure 2B,C). The development of microgametes could be divided into two phases: nuclear division of microgametocytes occurs first, and then the microgametes differentiate. The young microgametocyte bounded by a single-unit membrane contained several nuclei, in which some nuclei underwent mitosis and still possessed a nucleolus (Figure 2B). Subsequently, invaginations and fissures occurred in the surface area of the microgametocyte, which separated the microgametocyte into several parts or cytomere-like masses (Figure 2C,D). The nuclei migrated to the peripheral zone of the microgametocyte, where the mitochondria were located (Figure 2B,C). The nuclear chromatin was aggregated into the peripheral zone of each nucleus. Meanwhile, the entire nuclei began to protrude into PV or fissure (Figure 2C). Simultaneously, the centrioles appeared in the immediate vicinity of the nuclei, usually in the space between the nuclei and the surface of the microgametocytes or between the nuclei and the limiting membrane of the fissures (Figure 2B,C).

As development progressed, the protuberance on the surface of the gametocyte began to elongate, and the gamete nucleus, mitochondrion, and centriole were located in these protrusions (Figure 2E). By this stage, microgamete formation had been initiated by the growth of flagella, which protruded into the spaces around PV and inside the microgametocytes (Figure 2E,F). At the place of constriction between the microgamete and the cytoplasm of microgametocyte, the bounding membrane had an underlying cylinder-like osmiophilic layer (Figure 2E), in where the immature microgamete might be pinched off from the microgametocyte. The young microgamete separated from the cytoplasm of the gametocyte initially appeared as spherical or ovoid bodies that consisted chiefly of nuclear substance (Figure 2A,E,F).

Further development resulted in the maturation of microgamete (Figure 2E,F). The mature microgamete had a construction similar to that of other *Eimeria* species, with nucleus, mitochondria, and flagella (Figure 2F). The nucleus, which was membrane-bound (Figure 2F), occupied most of the space in the body of the microgamete; the mitochondrion was seen in close proximity to the nucleus near the apical pole of the microgamete; and the two gamete flagella, which contained the nine paired peripheral fibrils concentrically arranged around two central fibrils, were seen to arise from the basal apparatus (Figure 2G). In addition, an individual microtubule, which originated in the basal apparatus zone at the apical pole and extended over the area of the mitochondrion to the posterior end of the microgamete, was observed (Figure 2H).

### 3.3. Development and Ultrastructure of Macrogametocyte and Oocyst Wall Formation

#### 3.3.1. Macrogametocyte Development

**Figure 3:**
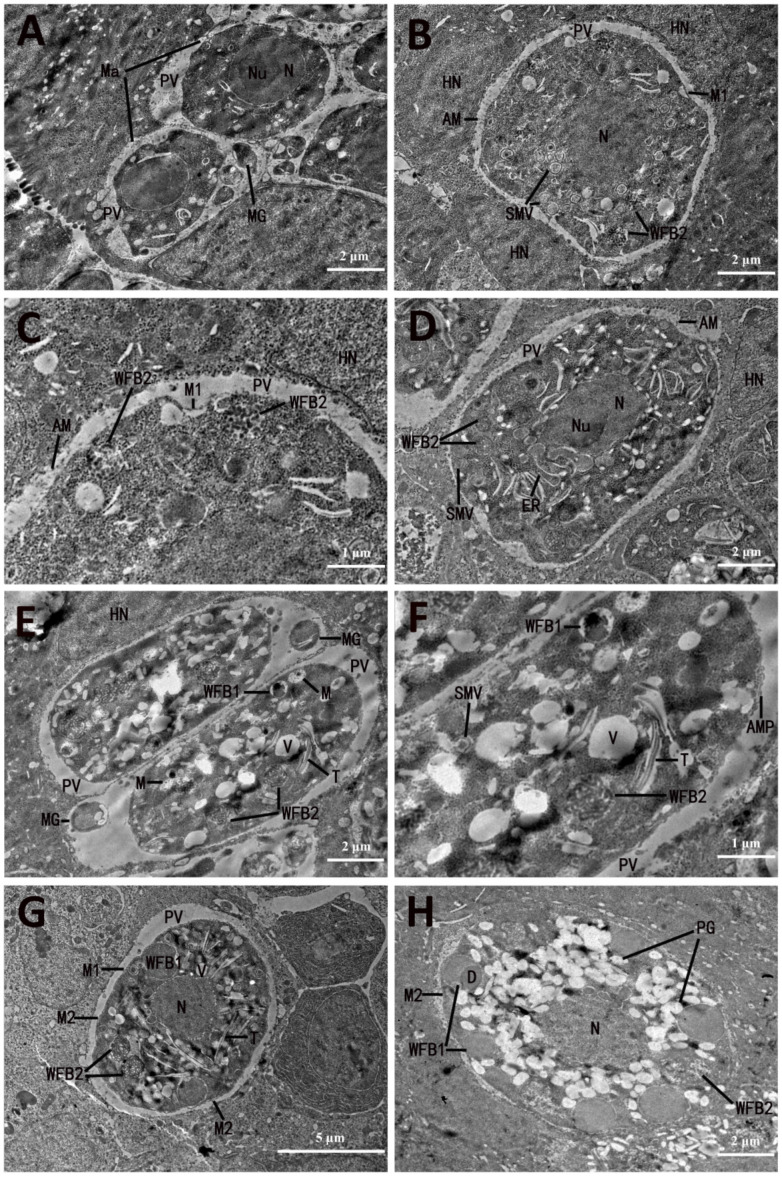
Electron micrographs of fine structures and development of macrogametocytes of *Eimeria necatrix*. (**A**) Early stage of Ma with the apical part of MG in the PV. (**B**) Young Ma with WFB2 and SMV in the cytoplasm. (**C**) An early stage of Ma with AM in PV. (**D**) An early stage of macrogametocyte with a central N and a Nu, and SMV, PG, WFB1, and WFB2 in the cytoplasm. (**E**) WFB1 is located in the outer region while WFB2 is in the deeper region of the cytoplasm, and numerous PGs appear after fertilization and SMV disappears. (**F**) Enlargement of the section displayed in (**E**) showing that AMP appeared on the surface of the gametocyte; (**G**) M1 and M2 formed, and the number of V and WFB2 gradually increased. The cytoplasm contained a large number of T. (**H**) Mature macrogametes showing the central N and a number of large peripherally located WFB1 intermixed with WFB2. The cytoplasm contains a large number of PGs. SMVs, small membrane-bounded vesicles; M, membrane; AMs, amorphous contents; N, nucleus; Nu, nucleolus; PV, parasitophorous vacuole; T, tubular structures; WFB2, wall-forming body 2; WFB1, wall-forming body 1; AMP, arch-shaped membrane protrusion; PGs, polysaccharide granules; OW, outer oocyst wall; V, vacuoles; L, lipid droplets; ER, endoplasmic reticulum; IW, inner oocyst wall; P, protoplast.

All stages of macrogametocyte development and oocyst wall formation were observed at 148 hpi, which was maintained within a PV limited at the host–cell boundary by an unclear unit membrane. The young macrogametocyte, which was surrounded by a cytoplasm limiting membrane (M1), possessed a large centrally located nucleus and a prominent granular nucleolus. The gametocyte cytoplasm was granular and included very few vacuoles (Figure 3A). As development progressed, a few small-membrane-bounded vesicles containing lucent amorphous contents or fine particles and early wall-forming body 2 (WFB2) appeared in the gametocyte cytoplasm (Figure 3B,C). At a slightly later stage, the number of membrane-bounded vesicles increased and migrated to the peripheral zone of the gametocyte. Simultaneously, a number of polysaccharide granules appeared in the gametocyte cytoplasm (Figure 3D). By this time, the macrogametocyte was itself limited by a unit membrane, although the surface structures were absent. Within the PV of the developing gametocyte, there were a few amorphous materials (Figure 3D).

As development proceeded, an arch-shaped membrane protrusion appeared on the surface of the gametocyte, and early wall-forming body 1 (WFB1) and tubular structures appeared in the cytoplasm of the gametocyte (Figure 3E,F). At this stage, a membrane-bounded body containing nuclear chromatin and mitochondrion, which were similar in appearance to cross-sections of the anterior region of a microgamete, appeared in the PV of the developing gametocyte (Figure 3D). As the macrogamete matured, the number of membrane-bounded bodies decreased and even disappeared. The arch-shaped membrane protrusion began to form an outer membrane named M2, and more tubular structures appeared in the cytoplasm surrounding the nucleus (Figure 3G). Concurrently, the number and size of the WFB1 increased and were located round the periphery of the gametocyte, and the cytoplasm contained a large number of tubular structures (Figure 3G). Mature macrogametes showed the central nucleus and a number of large peripherally located WFB1 intermixed with WFB2; the cytoplasm contained a large number of polysaccharide granules, and the membrane-bounded body disappeared (Figure 3H). The mature WFB1 had homogenous contents and appeared to be membrane-bound, and some of them contained a small inclusion body with a stronger osmiophilic appearance (Figure 3G). The mature WFB2 were less electron dense than WFB1 and had a loose, filamentous or sponge-like structure (Figure 3C,D,F). However, the contents in the early WFB2 appeared granular (Figure 3B). The number of WFB2 in the cisternae of the granular endoplasmic reticulum was generally one, but occasionally two (Figure 3D,F). In the mature macrogamete, the WFB2 formed a ring that was overlapping but internal to the ring formed by the WFB1 (Figure 3G).

#### 3.3.2. Oocyst Wall Formation

**Figure 4:**
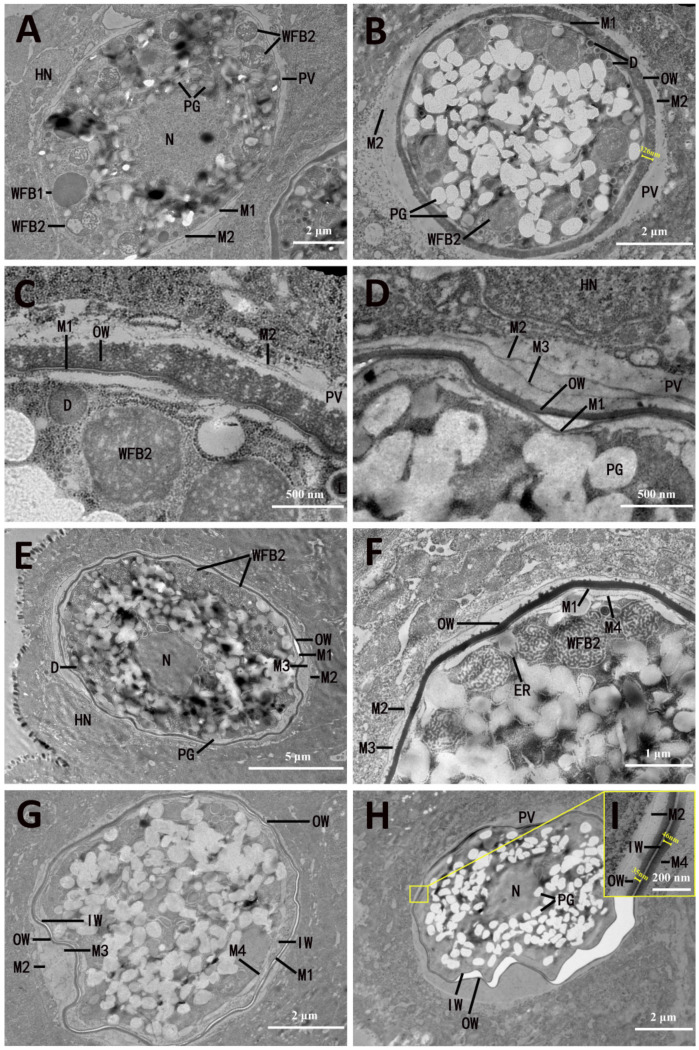
Electron micrographs of oocyst wall formation of *Eimeria necatrix*. (**A**) The WFB2 moves to line up below the M1; (**B**) WFB1 fused to form the OW between M1 and M2; (**C**) enlargement of the section displayed in (**B**) showing that OW formed while D remained; (**D**) the thickness of OW decreases, and the M3 appears; (**E**) WFB2 arrange around the periphery of the Ma; (**F**) WFB2 discharges its contents between M1 and M4; (**G**) WFB2 is released on the inner side of OW to form IW and disappears from the cytoplasm. (**H**) Fully formed oocysts contained a large central N and numerous PGs; (**I**) enlargement of the section displayed in (**H**) showing the thickness of OW and IW. N, nucleus; Nu, nucleolus; M, membrane; PV, parasitophorous vacuole; WFB2, wall-forming body 2; WFB1, wall-forming body 1; PGs, polysaccharide granules; OW, outer oocyst wall; V, vacuoles; ER, endoplasmic reticulum; IW, inner oocyst wall.

This commenced by the fusion of a number of interrupted membrane portions to form a loose veil (M2) around the parasite (Figure 4A). As the M2 separated from the M1, an empty space between the M1 and M2 was formed (Figure 4A). Then, the WFB1 migrated into the space between the M1 and M2 and released their contents, which fused to form the outer oocyst wall (Figure 4B). The WFB1 material initially formed an uneven 350–750 nm thick layer (Figure 4B,C), and then appeared to polymerize to form the outer layer of the oocyst wall, which was composed of two parts: an amorphous exterior and an osmiophilic interior. The osmiophilic part was approximately 45 nm thick with a granular coat (Figure 4D,I). During this process, there was evidence of a new membrane (M3) formed below the M2 (Figure 4C,D), which might derive from the membrane of WFB1. By the time the WFB1 disappeared, some small dense granules of WFB1 remained (Figure 4B,C).

**Figure 5:**
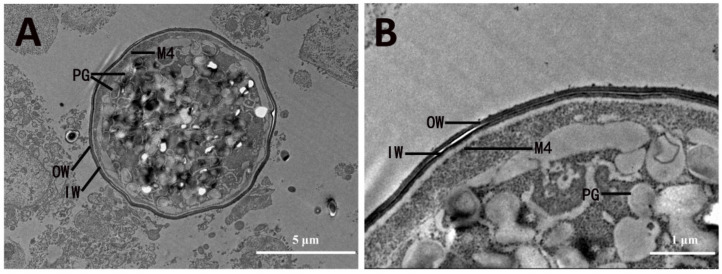
Electron micrographs of oocyst wall of *Eimeria necatrix*. (**A**) The young oocyst broke out of the host cell; (**B**) enlargement of the section displayed in (**A**) showing the oocyst released into the external environment remained with OW, IW, and M4. OW, outer oocyst wall; IW, inner oocyst wall; M, membrane.

Formation of the inner layer of the oocyst wall commenced with the completion of the outer layer of the oocyst wall. During this process, there was aggregation of the WFB2 around the periphery of the parasite (Figure 4E). Concurrent with this was the formation of another inner limiting membrane (M4) beneath M1 (Figure 4F). As development proceeded, the WFB2 began to discharge its contents internal to M1, but external to M4 (Figure 4F), in which the WFB2 material polymerized to form the inner layer of the oocyst wall (Figure 4G), approximately 46 nm thick (Figure 4F). After the formation of the inner layer of the oocyst wall, M4 became the cytoplasmic membrane of the sporont. Fully formed oocysts sometimes had an irregular perimeter and contained a large central nucleus and numerous polysaccharide granules, in which no wall-forming bodies were present (Figure 4F). As the young oocyst broke out of the host cell (Figure 5A), its sporont appeared contracted, which resulted in a gap between the oocyst wall and the sporont (Figure 5B).

### 3.4. Ultrastructural Localization of EnGAM22 and EnGAM59 Proteins

**Figure 6:**
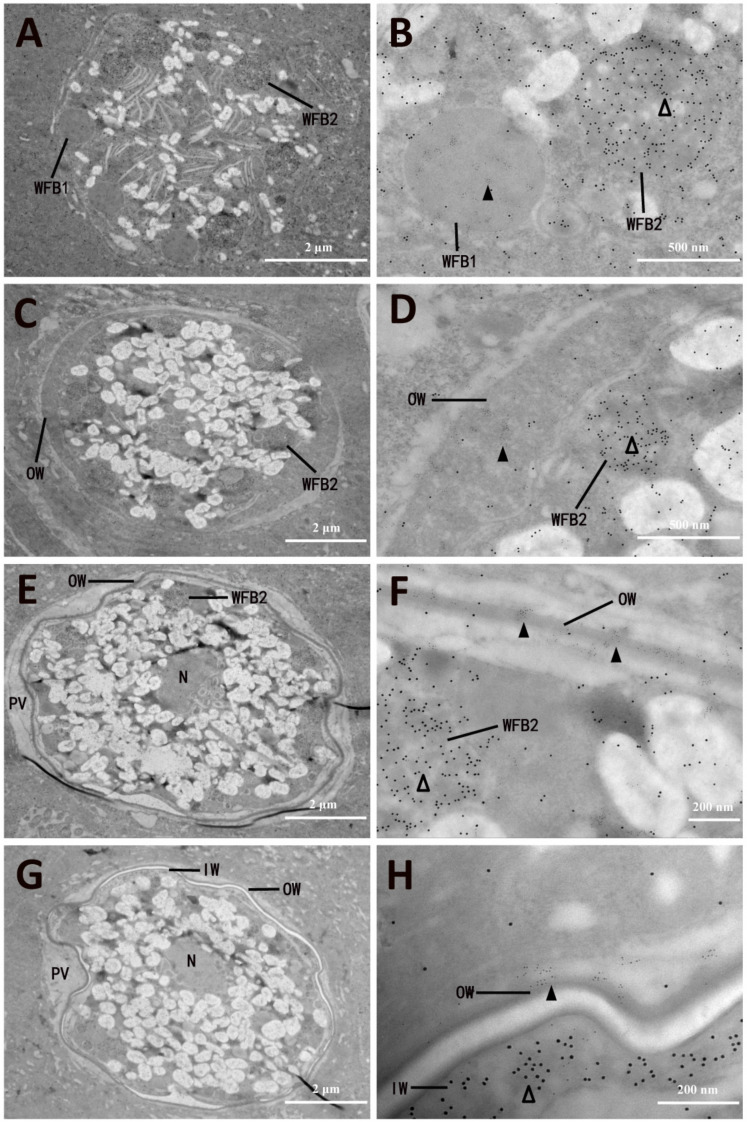
Immunoelectron micrographs of macrogametocytes of *Eimeria necatrix*. (**A**–**H**) Immunoelectron microscopy co-localization of EnGAM22 and EnGAM59 in macrogametocytes incubated with rabbit anti-rEnGAM22 pAb (visualized with 4 nm golden particles) and mouse anti-rEnGAM59 pAb (visualized with 12 nm golden particles). (**A**) An early-stage Ma displaying multiple WFB1 and WFB2 dispersed throughout the cytoplasm. (**B**) Enlargement of the section displayed in (**A**) showing EnGAM22 were located in WFB1 (▲) while EnGAM59 were located in WFB2 (△). (**C**) The slightly later stage of Ma contained initially formed OW and WFB2. (**D**) Enlargement of the section displayed in (**C**) showing EnGAM22 were located in OW (▲). (**E**) The fully formed OW in the Ma. (**F**) Enlargement of the section displayed in (**E**) showing EnGAM22 were located in OW (▲). (**G**) The late stage of Ma contained OW and IW. (**H**) Enlargement of the section displayed in (**G**) showing EnGAM59 were located in IW (△). WFB2, wall-forming body 2; WFB1, wall-forming body 1; OW, outer oocyst wall; IW, inner oocyst wall.

The ultrastructural localization of EnGAM22 and EnGAM59 proteins in the mature macrogamete and formation of the oocyst wall of *E. necatrix* were observed using immuno-electron microscopy, in which the tissue samples were co-labeled with the rabbit anti-rEnGAM22 pAb and mouse anti-rEnGAM59 pAb together, and then visualized using goat anti-rabbit Ig conjugated to 4 nm gold particles and goat anti-mouse Ig conjugated to 12 nm gold particles. The results showed that the WFB1 in the mature macrogamete was specifically labeled with numbers of 4 nm gold particles (EnGAM22-proteins), whereas WFB2 was specifically labeled with numerous 12 nm gold particles (EnGAM59-proteins) (Figure 6A,B). As the fusion of the WFB1 at the surface of the parasite and the release of their contents, the outer layer of the oocyst wall was formed. By this time, the 4 nm gold particles were specifically distributed in the outer layer, while the 12 nm gold particles were specifically distributed in the WFB2 (Figure 6C–F). As the completion of the outer layer, the WFB2 beneath M2 began to discharge its contents, which polymerized to form the inner layer. By this time, the 12 nm gold particles were specifically distributed in the WFB2 and the inner layer, while the 4 nm gold particles were specifically distributed in the outer layer (Figure 6E–H).

## 4. Discussion

The life cycles of normal strains of *E. necatrix* consist of three generations of meronts, and their third-generation meronts occupy enterocytes in the surface epithelium or crypts of the caeca, containing up to 16 merozoites [14,16]. In the present study, eight MZ-3 were observed, and their ultrastructure was similar to that of second-generation merozoites of *E. necatrix* [14] and other *Eimeria* species [17,18]. Apart from possessing classical features and organelles of typical eukaryotic cells such as the nucleus, mitochondrion, and endoplasmic reticulum, the MZ-3 of *E. necatrix* contained several features specific to the phylum, such as conoid, rhoptries rhoptry, micronemes, and dense granule. However, further research is needed to determine whether MZ-3 of *E. necatrix* are developed by a surface ‘budding’ mechanism as in *Eimeria tenella* [17] rather than by an internal splitting of the schizont cytoplasm in *Eimeria perforans* and *Eimeria stiedae* [19].

The fine structure and development of *E. necatrix* microgametes were similar to earlier descriptions [20] and that of other *Eimeria* species [17,21,22,23,24,25,26]. The microgametogenesis of *E. necatrix* was divided into two phases: nuclear proliferation and separation of the numerous microgametes from the microgametophytes. The mature microgametes primarily consisted of the nucleus, a mitochondrion, flagellar structures, and a basal apparatus. In the process of microgametogenesis of *E. necatrix*, the emergence of the developed flagella preceded that of the microgamete body, which was consistent with that of *E. maxima* [27], *E. magna* [28], *E. brunetti* [29], and *E. tenella* [30]. Only a portion of the karyoplasm was incorporated into the nucleus of the microgamete, which differed from the situation in rabbit coccidia *E. perforans* [21], where all the karyoplasm was integrated. Each microgamete of *E. necatrix* had a mitochondrion that was closely associated with the nucleus and two flagella that appeared to join the body at the apex. These observations were at variance with that of Regal [20], who reported that microgametes of *E. necatrix* had three flagella. The final number of microgametes produced from a single microgametocyte was not able to be confirmed in this study.

The present observations showed that the development of *E. necatrix* macrogametocyte could be divided into three phases: the WFB2 develops; the WFB1 and polysaccharide granules develop; and the WFB1 and WFB2 appear as a concentric arrangement around the nucleus. The size of WFB1 was larger than that of WFB2. The mature WFB1 with homogenous contents was bounded by a layer of membrane, whereas the mature WFB2 with less electron density had a loose, filamentous, or sponge-like structure. These findings were basically consistent with the reports by Dubremetz and Yvore [13] and Regal [20,31], and similar to that of other *Eimeria* species as described by Scholtyseck et al. [32], Michael [33], Ferguson et al. [6], Kefu et al. [34], and Wiedmer [35]. But no intravacuolar tubules and micropores were observed in the present study, which differed from the findings of Dubremetz and Yvore [13], who observed large numbers of tubules at the surface of the macrogametocyte and a micropore at the surface of the macrogamete during the formation of the oocyst wall. However, we found large numbers of the arch-shaped membrane protrusion at the surface of the developing macrogametocyte and a large amount of small-membrane-bounded vesicles in the cytoplasm of early developmental stages of the macrogametocyte, respectively. These vesicles, which were no longer present after the maturation of the macrogamete, might be the same organelle as the electron-pale vesicles described in *E. maxima* by Pittilo and Ball [36], later termed veil-forming bodies by Ferguson et al. [6]. We suggested that these small vesicles are released to the cell surface, and then their membranes are connected to each other to form a veil surrounding the macrogamete.

As other *Eimeria* spp., the oocyst wall formation of *E. necatrix* commenced with formation of a loose surrounding veil. But the origin of the veil differed from other *Eimeria* species or other authors’ descriptions. In *E. brunetti*, a mature macrogamete (or zygote) surrounded by an amorphous layer possessed a single outer unit membrane, plus two closely applied inner unit membranes, and its veil, which consist of the amorphous layer and the two other membranes and are formed via separation of the two closely applied inner membranes [37,38]. In *E. maxima*, a mature macrogamete possessed two limiting membranes underlying an interrupted third membrane, and its veil was formed by the outer unit membrane with a separation from the inner unit membrane [6,39]. Similar to *E. maxima*, a mature macrogamete of *E. acervulina* had two outer membranes (m1 and m2) and an amount of interrupted membrane portions (m3) just beneath the outer membrane, and its veil was formed by the m1 with a separation from the m2 [40]. In the present study, the mature macrogamete of *E. necatrix* had two limiting membranes (M1 and M2), and the M2 originating from the fusion of an amount of interrupted membrane portions separated from the M1 to form a loose veil. These observations differed from that of [13], who found that a mature macrogamete of *E. necatrix* surrounded by a ring of hemispherical microtubules had a limiting membrane. As development proceeded, the hemispherical microtubules at the surface of the macrogamete disappeared. Concurrent with this was the appearance of another two membranes. The outer membranes were separated from the inner membrane to form a gap.

It is generally accepted that the oocyst wall formation in coccidia does not begin until after fertilization of the mature macrogamete, although little is known concerning the process of fertilization in coccidia. Scholtyseck [41] observed a microgamete at the nuclear envelope of the macrogamete of *E. maxima*. Scholtyseck and Hammond [42] found that the entire microgamete, including two flagella, entered the macrogamete. Using the scanning electron microscope, Madden and Vetterling [30] observed a macrogamete of *E. tenella* surrounded by adhering microgametes. Elwasila [43] showed that the microgametes of *E. maxima* approached the macrogamete with their nuclei directed forward and that the entire microgamete with its two flagella, mitochondrion, and microtubules was in contact with the macrogamete. Disappointingly, fertilization was not observed in the present study. However, we observed that when the arch-shaped membrane protrusion appeared on the surface of *E. necatrix* gametocyte, the anterior end of a microgamete without flagella appeared in the PV of macrogametocyte. And when the interrupted membrane portions formed a loose veil (M2) around the gametocyte, the anterior end of a microgamete disappeared in the PV. This founding confirmed that fertilization of *Eimeria* species macrogametes precedes oocyst wall formation.

With the formation of a loose surrounding veil in *E. necatrix*, the WFB1 was transferred to the periphery of the macrogamete and fused together to form the outer layer of the oocyst wall; shortly after this, the WFB2 was transferred to the periphery and fused together, forming the inner oocyst wall. These findings were basically consistent with the observations of Dubremetz and Yvore [13] and Regal [31] in *E. necatrix*, but they did not describe the details of these process, particularly with regard to formation of the inner oocyst wall. In the present study, we found that the WFB1 was transferred to the space between the veil (M1) and M2 and fused with each other; then, the contents of the WFB1 polymerized and concentrated to form the outer layer of the oocyst wall, and concurrent with this was that the membrane of the WFB1 formed M3 beneath M1. Shortly after the completion of the outer layer, another inner limiting membrane (M4) began to form beneath M1; then, the WFB2 fused with the M4 to discharge its contents external to M4 but internal to M1; and finally, the contents of WFB2 polymerized to form the inner layer of the oocyst wall, with M4 becoming the cytoplasmic membrane of the sporont in the meantime. These findings were consistent with the observations of McLaren in *E. tenella* [17], Ferguson et al. in *E. brunetti* [37], Michael in *E. acervulina* [33], and Ferguson et al. in *E. maxima* [6], although the number of membranes that appeared during oocyst wall formation varied between species but differed from those of Pittlo and Ball [40], who found that the WFB2 in *E. acervulina* appeared to discharge their contents between the cytoplasmic limiting membranes (M2 and M3).

The oocyst wall of mature oocysts of *E. necatrix* in host cells was double-layered. The outer layer possessed an amorphous exterior and an osmiophilic interior and measured 35 nm in thickness. The inner layer had a uniform width of 46 nm in thickness. The wall of oocysts liberated from the host cells was similar to that of the oocysts within the host cells. But the veil and amorphous exterior of the outer layer disappeared. Concurrent with this was that the cytoplasm bounded by a single unit membrane had shrunk away from the oocyst wall. These findings were consistent with the observations in *E. maxima* reported by Pittlo and Ball [39] but differed from the observations in *E. tenella* reported by McLaren [17], who found the oocyst wall of *E. tenella* remained trilaminate, even after the oocyst was liberated into the cecal lumen.

Localization of specific proteins within cells at the nanometer level of resolution is central to understanding how these proteins function in cell processes such as trafficking. The immunogold electron microscopic co-localization technique can be used to determine the localization of EnGAM22 and EnGAM59 proteins in the gametocyte and oocyst wall of *E. necatrix* [44]. The cecal tissue sections were incubated with a rabbit anti-rEnGAM22 and mouse anti-rEnGAM59 primary antibody mixture, and the primary antibodies were visualized with 4 nm gold-conjugated goat anti-rabbit IgG and 12 nm gold-conjugated goat anti-mouse anti-rabbit IgG. The results showed that the 4 nm gold particles labeling EnGAM22 protein mainly localized to the WFB1 and the outer layer of the oocyst wall, whereas 12 nm gold particles labeling EnGAM59 protein mainly localized to the WFB2 and the inner layer of the oocyst wall. These results were consistent with the observations of our previous study using an indirect immunofluorescence assay and laser confocal microscopy [9], further confirming that EnGAM22 and EnGAM59 proteins are the structural components of the oocyst wall of *E. necatrix*. However, how EnGAM22 and EnGAM59 proteins form the oocyst wall needs further investigation.

## 5. Conclusions

This study focused on the ultrastructural changes occurred during the third merogony, microgametogenesis, and macrogametogenesis of *E. necatrix*. The results showed that the third-generation meront contained eight merozoites. The fine structure and developmental processes of merozoites, microgametes, and macrogametes were similar to those of other *Eimeria* species except for two flagella in microgametes and no intravacuolar tubules and micropores in macrogametes. The process of oocyst wall formation of *E. necatrix* was concurrent with that of other *Eimeria* species. There are four membranes participating in the formation of the oocyst wall and the veil surrounding the macrogamete originates from the small-membrane-bounded vesicles. EnGAM22 and EnGAM59 proteins are the structural components of the oocyst wall of *E. necatrix*. This study provides basic knowledge about the fine structure and development of *E. necatrix*, which may represent targets for counteracting parasite transmission.

## Data Availability

The raw data supporting the conclusions of this article will be made available by the authors on request.
